# Supplementary material for: Large-scale physically accurate modelling of real proton exchange membrane fuel cell with deep learning
Source: Nat Commun. 2023 Feb 14;14:745. doi: 10.1038/s41467-023-35973-8 (PMC9929041; doi:10.1038/s41467-023-35973-8)
Supplement: Supplementary file 1 — Supplementary Information [file 41467_2023_35973_MOESM1_ESM.pdf]

Supplementary Materials to Large-scale Physically Accurate modelling of Real Proton Exchange Membrane Fuel Cell with Deep Learning

Ying Da Wang *[a], Quentin Meyer †[b], Kunning Tang[a], James E. McClure[c], Robin T. White[d], Stephen Kelly[d], Matthew Crawford[e], Francesco Iacoviello[f], Dan Brett[f], Paul Shearing[f], Peyman Mostaghimi[a], Chuan Zhao ‡[b] and Ryan T. Armstrong §[a]

[a] *School of Minerals and Energy Resources Engineering, University of New South Wales, Sydney, NSW, 2052, Australia*

[b] *School of Chemistry, University of New South Wales, Sydney, NSW, 2052, Australia*

[c] *National Security Institute, Virginia Tech, Blacksburg, VA, 24061, USA*

[d] *Carl Zeiss X-ray Microscopy, Dublin, CA, 94568, USA*

[e] *Fuel Cell Store, College Station, TX, 77845, USA*

[f] *Electrochemical Innovation Lab, Department of Chemical Engineering, University College London, London WC1E 7JE, United Kingdom*

Nature Communications

*These authors contributed equally. Email: yingda.wang@unsw.edu.au
†These authors contributed equally. Email: q.meyer@unsw.edu.au
‡These authors jointly supervised this work. Email: chuan.zhao@unsw.edu.au
§These authors jointly supervised this work. Email: ryan.armstrong@unsw.edu.au

1 Supplementary Table 1: Imaging Comparison of PEMFC Studies

Field of View	Voxel Resolution	Pixels per slice	Reference
0.7 × 0.7 mm	690 nm	1,015 × 1,015	[28]
0.7 × 0.5 mm	800 nm	875 × 625	[29]
0.7 × 0.7 mm	780 nm	900 × 900	[30]
1.6 × 1.6 mm	1.6 μm	1,000 × 1,000	[31]
1.5 × 1.5 mm	1.48 μm	1,015 × 1,015	[32]
3.0 × 3.0 mm	1.6 μm	1,875 × 1,875	[33]
2.4 × 2.4 mm	1.33 μm	1,875 × 1,875	[34]
6.5 × 6.5 mm	3.25 μm	2,000 × 2,000	[35]
3.8 × 3.8 mm	1.85 μm	2,045 × 2,045	[36]
3.3 × 3.3 mm	1.55 μm	2,200 × 2,200	[37]
2.2 × 2.2 mm	960 nm	2,304 × 2,304	[39]
8.8 × 5.9 mm	2.2 μm	4,000 × 2,681	[38]
5.6 × 2.8 mm	2.8 μm	2,000 × 1,000	This study, original low-resolution image
**5.6 × 2.8 mm**	**700 nm**	**8,000 × 4,000**	**This study, super-resolved image**

Table 1a: Comparison of the μ-CT image field of view, resolution, and digital size of PEMFCs in recent studies.

Field of View	Voxel Resolution	Domain (voxels)	LBM Method	Ref
50^3 μm	1.0 μm	50^3	3D Single-phase	[57]
80^3 μm	1.6 μm	50^3	3D Two-phase	[63]
125 × 125 × 360 μm	2.5 μm	50 × 50 × 144	3D Two-phase	[64]
1 × 0.4 mm	1.0 μm	1,000 × 400	2D Two-phase	[65]
500 × 200 μm	500 nm	1,000 × 400	2D Two-phase	[66]
150 × 192 × 480 μm	960 nm	156 × 200 × 500	Two-phase	[39]
623 × 271 × 264 μm	1.76 μm	354 × 154 × 150	Single-phase	[58]
3.9 × 3 × 0.875 mm	5.0 μm	780 × 600 × 175	3D Two-phase w/phase change	[69]
1.2 × 1.2 × 0.28 mm	1.3 μm	923 × 923 × 215	3D Single-phase	[67]
4 × 3 × 0.5 mm	2.9 μm	1,440 × 1,080 × 186	Single-phase	[56]
**5.6 × 2.8 × 0.54 mm**	**700 nm**	**8,000 × 4,000 × 775**	**3D Two-phase**	**This study**

Table 1b: Comparison of the field of view, resolution, simulated domain size, and simulation method of PEMFCs in recent studies.

2 Supplementary Figure 1: Training and Validation Comparison of DualEDSR and 3D-EDSR

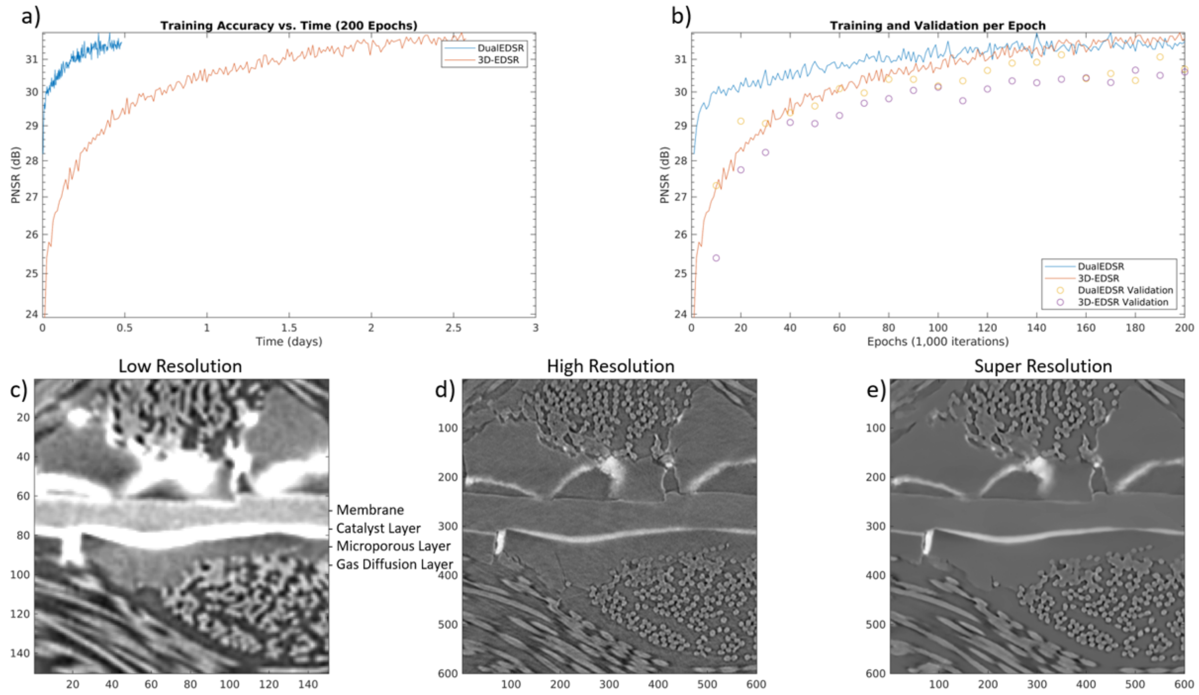

Figure 1: Graphs and visual comparisons of (a) training time and (b) validation performance comparing 3D-EDSR and DualEDSR. DualEDSR achieves a higher training and validation accuracy while also being 5 times faster during training. Visual comparison of a slice (a-c) from the validation set, with the greyscale as-yet unsegmented layers annotated. The super-resolved image shows typical noise reduction and image quality improvement from SRCNNs, and facilitates the subsequent accurate segmentation of the PEMFC.

3 Supplementary Figure 2: 3D Render of the Super Resolved Greyscale Domain

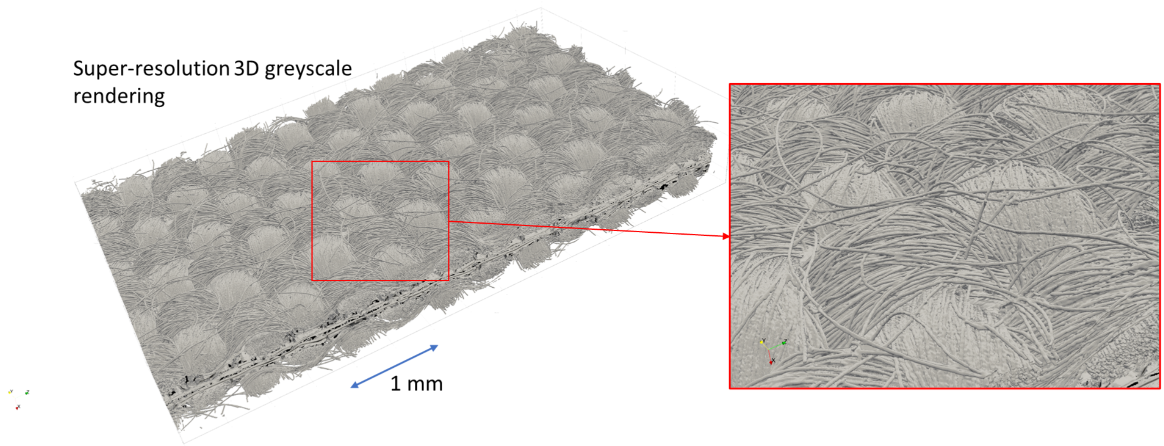

Figure 2: 3D render of the super resolved image

4 Supplementary Figure 3: Super Resolution Errors due to Out of Domain Image Artifacts

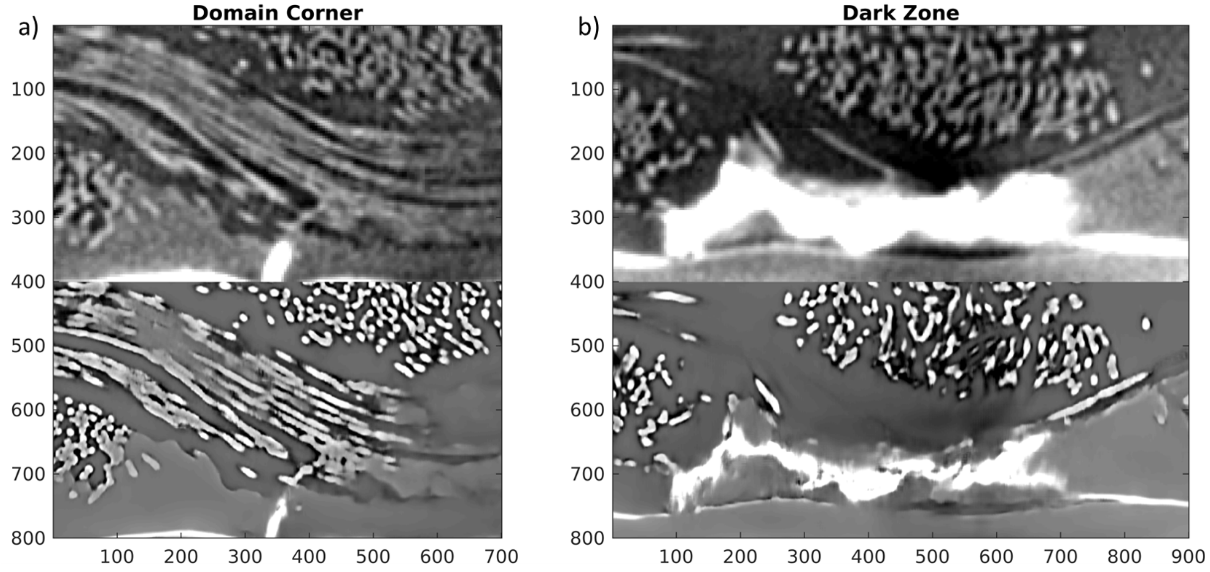

Figure 3: Examples of super-resolved errors in the image due to out-of-domain errors associated with CT image variations in the low-resolution image not included in the training data. (a) Dark regions at the corner of the image, and (b) dark regions adjacent to very bright catalyst layer bodies.

5 Supplementary Figure 4: Segmentation Training and Accuracy

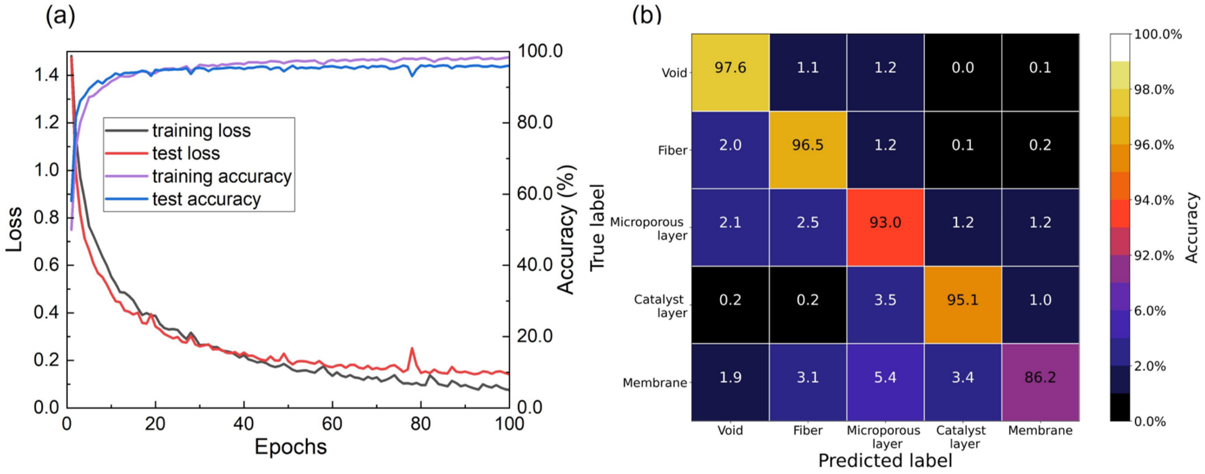

Figure 4: Segmentation training and accuracy, with (a) CNN training and testing loss and accuracy for 100 epochs, and (b) confusion matrix of the testing data. Y axis refers to the ground truth, X axis refers to the prediction by CNN. Diagonal values refer to the corrected labeled pixels.

6 Supplementary Figure 5: Super Resolved and Multi-Label Segmented PEMFC Cross Sections

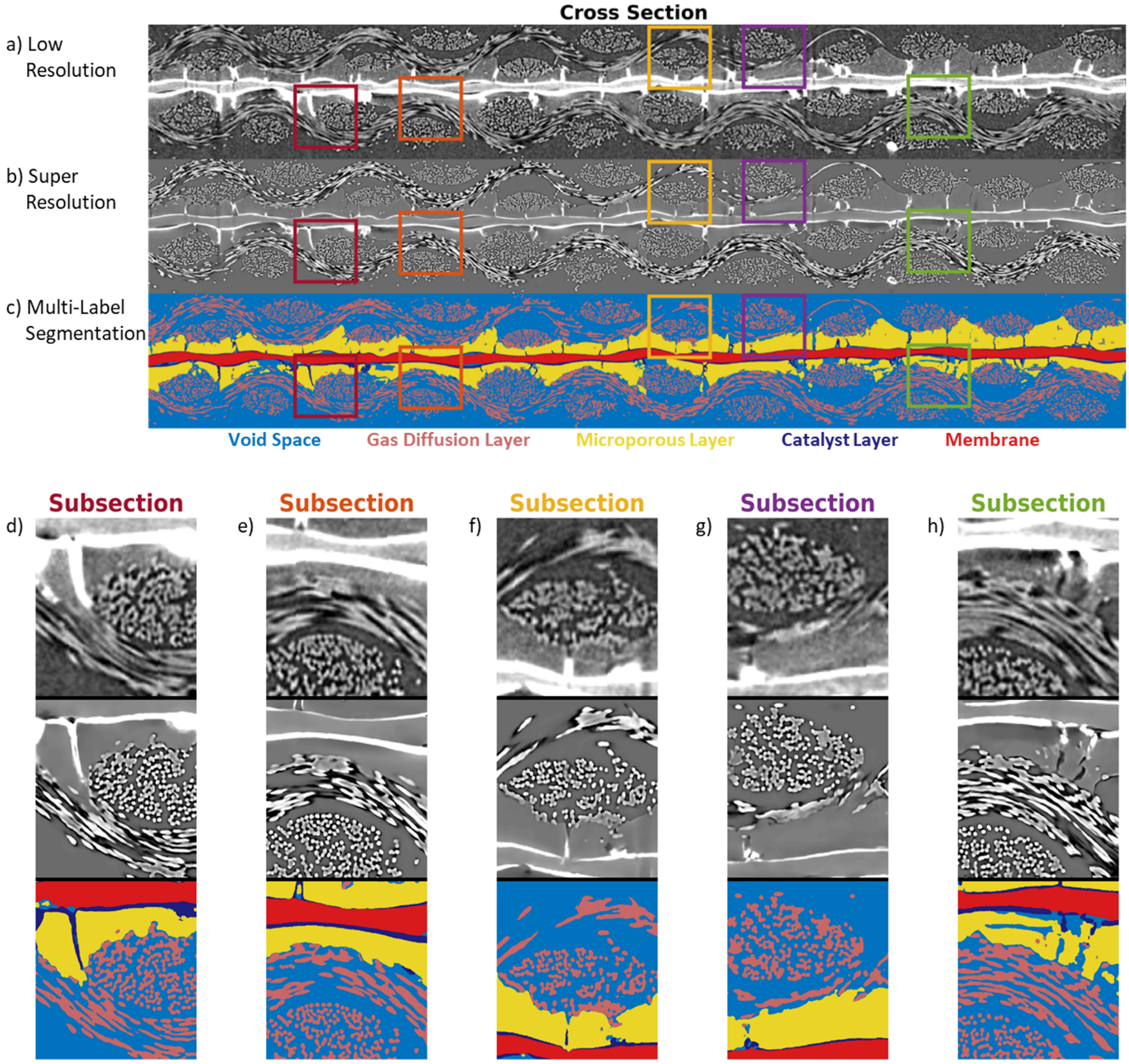

.

Figure 5: Comparison with selected zoomed in subsections of a) 2D cross section of the low-resolution for comparison with (b) the super-resolved image and (c) multi-label segmented image. The improvement in image quality and resolution can be clearly seen over the low-resolution image, and permits the accurate identification and delineation of membrane, catalyst layer, MPL, and GDL

7 Supplementary Figure 6: Comparing Segmentation Accuracy between Manual, Low-Resolution CNN, and Super-Resolution CNN

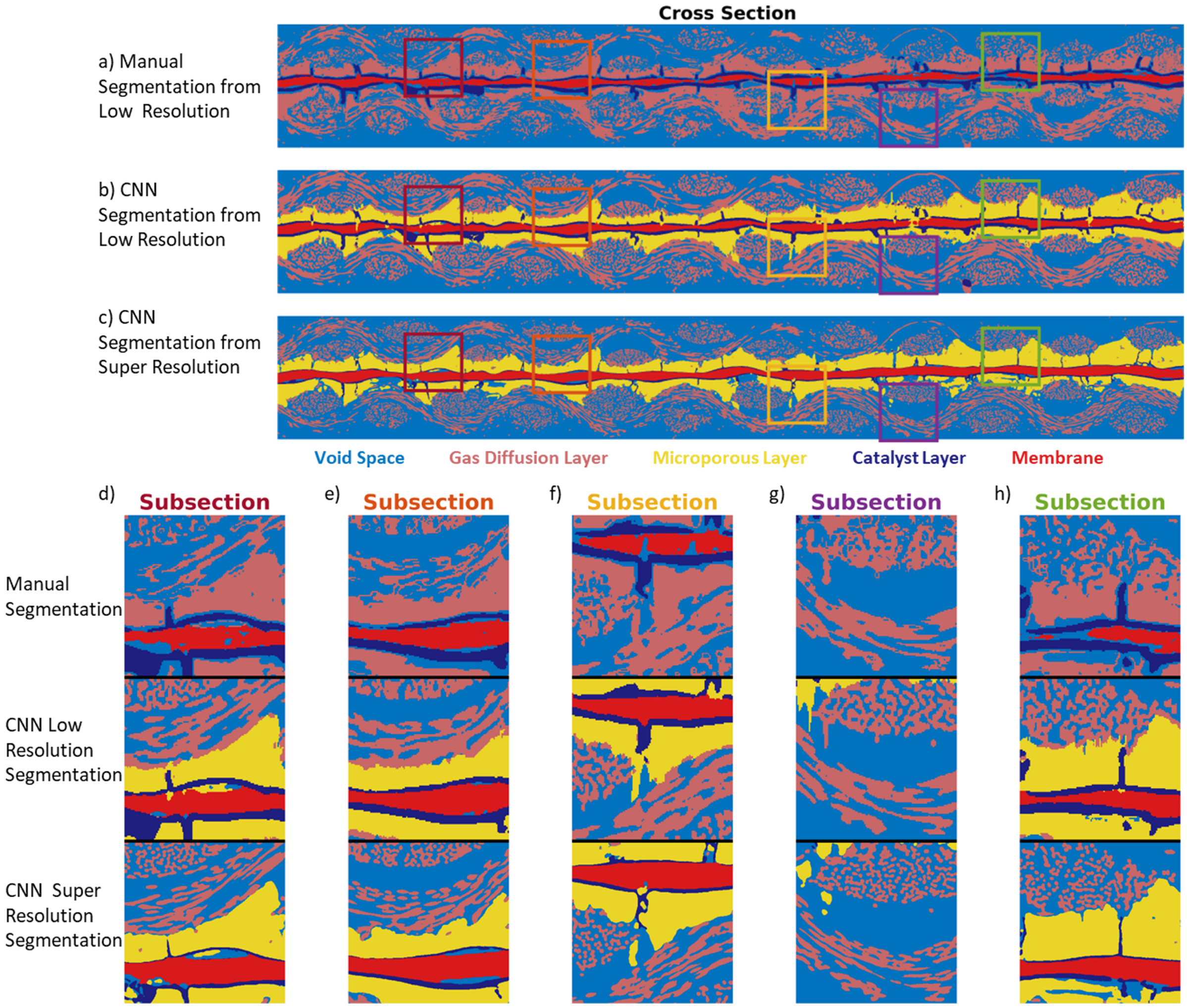

Figure 6: Comparison with selected zoomed in subsections of a) 2D cross section of manual segmentation using Avizo software on the low-resolution μ-CT image for comparison with (b) multi-label CNN segmentation on the low-resolution μ-CT image and (c) multi-label segmented and super-resolved image. The manual segmentation failed to identify the MPL from the GDL, and both segmentations on the low-resolution image suffer significantly from blur and diffuse boundaries, resulting in oversized catalyst thickness and inaccurate MPL and GDL fiber geometries.

8 Supplementary Figure 7: Full Volume Renders of Individual Segmented PEMFC Layers

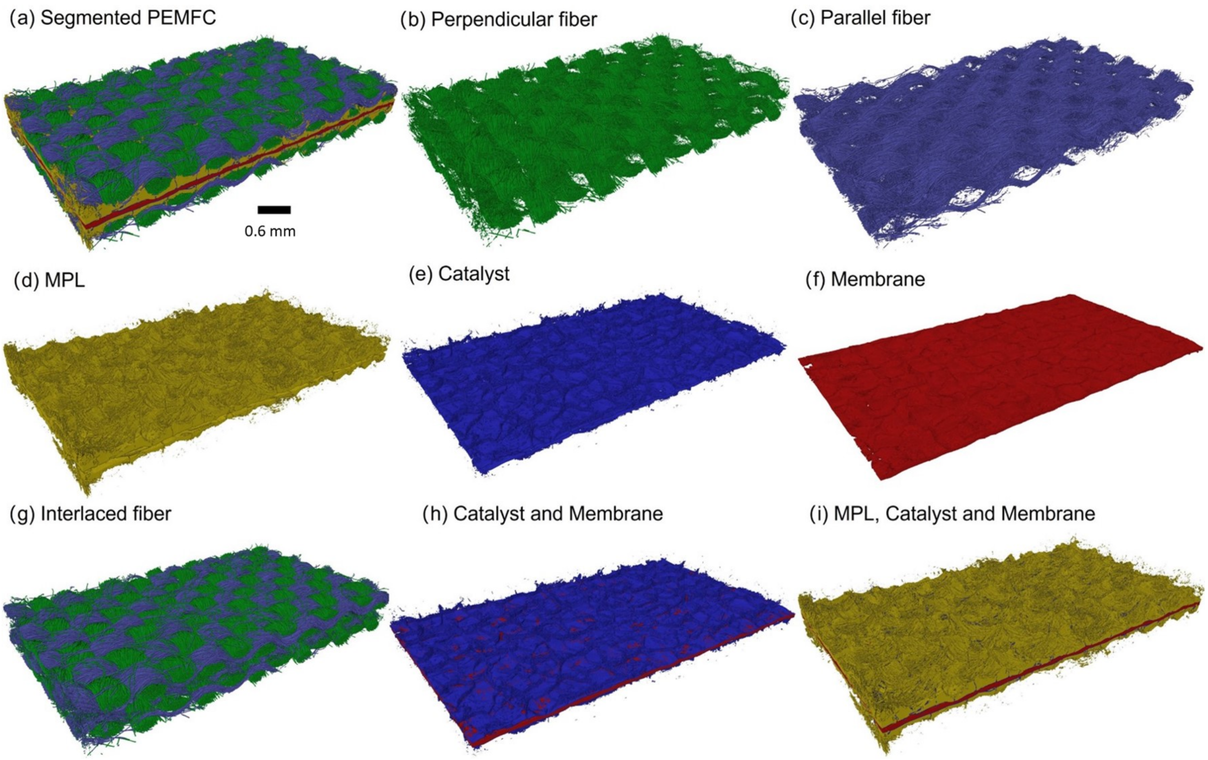

Figure 7: 3D volume rendering of the segmented PEMFC and the different materials. (a) full feature segmentation of PEMFC. (b) Perpendicular fiber. (c) Parallel fiber. (d) MPL. (e) Catalyst. (f) Membrane. (g) Interlaced fiber. (h) Membrane with catalyst at top and bottom. (i) Membrane and catalyst with MPL deposited at top and bottom.

9 Supplementary Figure 8: Segmentation Errors

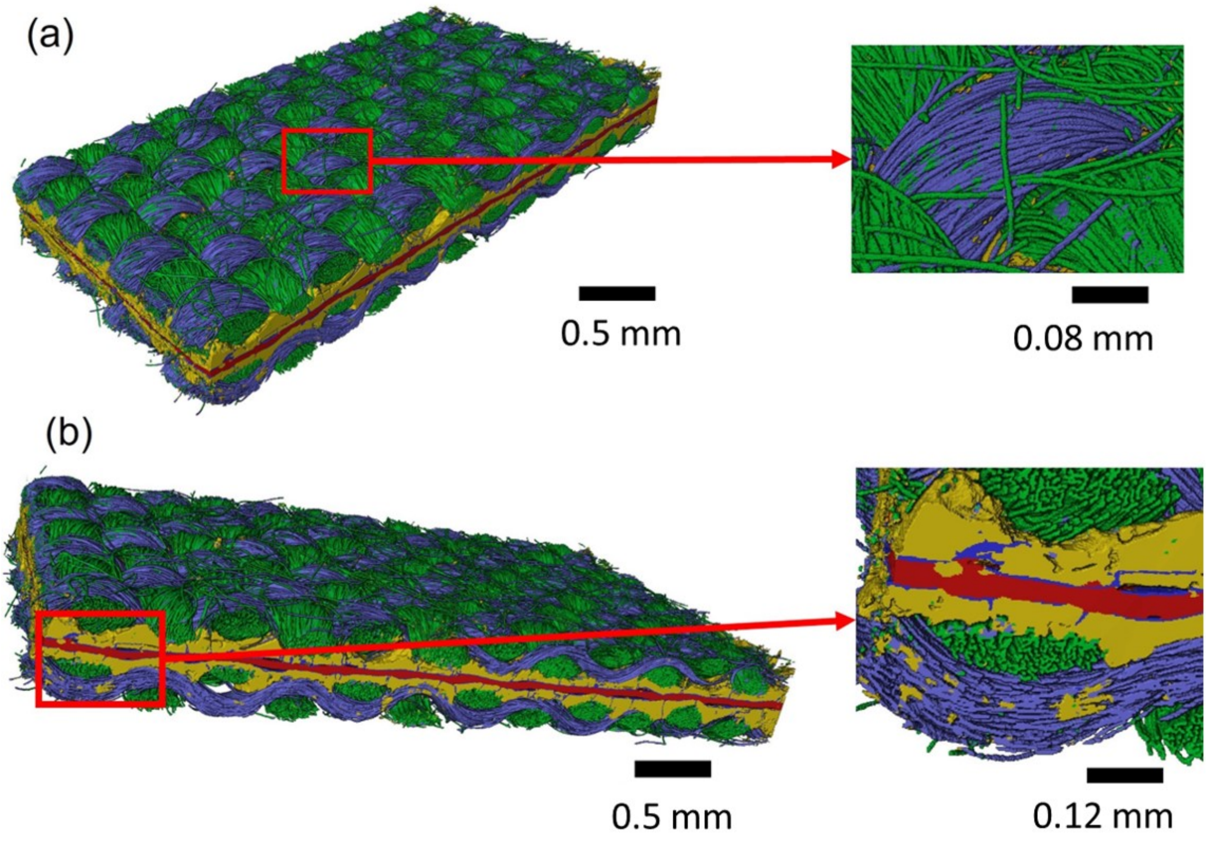

Figure 8: Visualisation of the error regions in the segmentation. (a) Error between the perpendicular fiber and parallel fiber. (b) Error of mislabeling the membrane and parallel fiber as MPL due to the similar voxel intensity.

10 Supplementary Figure 9: Renders of Wide Scale Heterogeneity in MPL and GDL

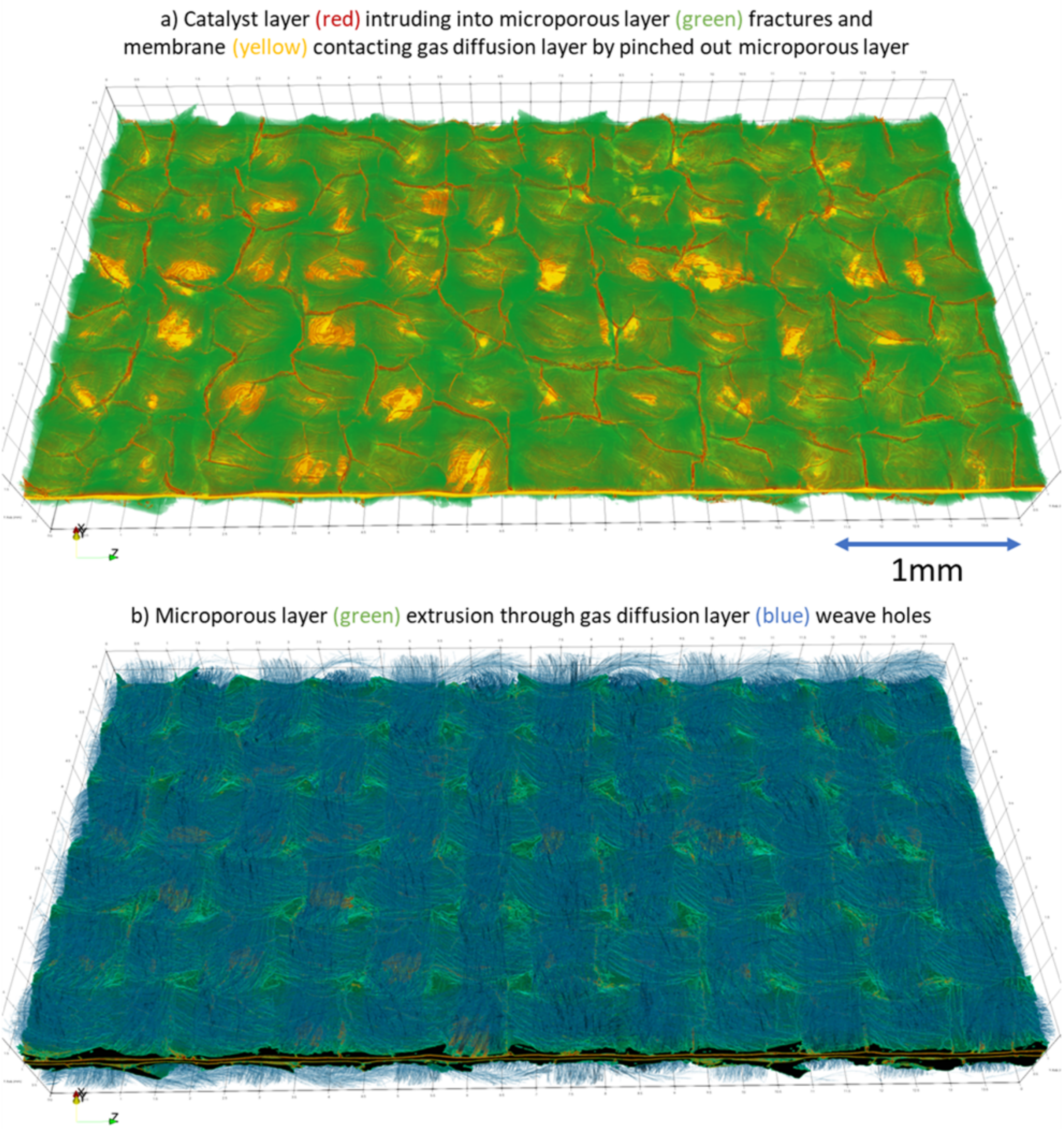

Figure 9: Renders of the heterogeneity observed at wide field-of-view length scales. (a) Shows that a fracture network in the MPL exists where the catalyst layer has extruded into and also being pinched thinly towards the catalyst layer by the GDL weave, and (b) shows the MPL itself has conformed to the GDL weave by extruding out through the holes on the weave.

11 Supplementary Figure 10: Velocity Field Heterogeneity in PEMFC

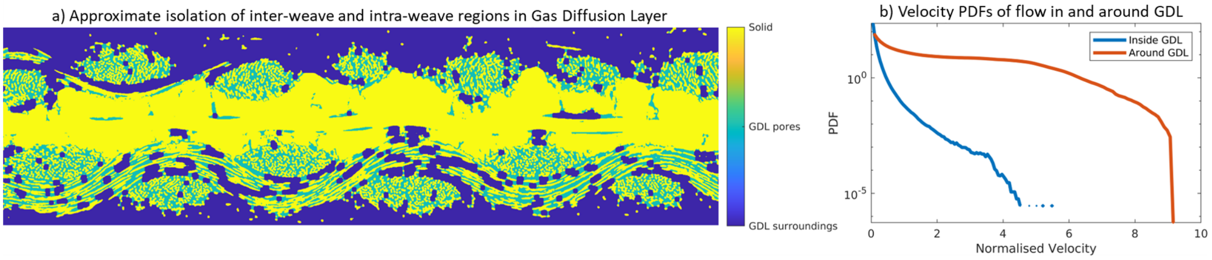

Figure 10: (a) Regions within and around the GDL, as approximated by morphological closing operations allow analysis of (b) Velocity PDFs $\frac{|\bar{v}|}{|\frac{1}{V}\int_V \bar{v} dV|}$ of flow within and around the GDL. The magnitude difference between the regions is clear and shows that a significantly higher fraction (>10x) of the GDL surroundings flows at a higher normalised velocity of 2-4, reaching up to 8.

12 Supplementary Figure 11: Velocity Field Heterogeneity in PEMFC

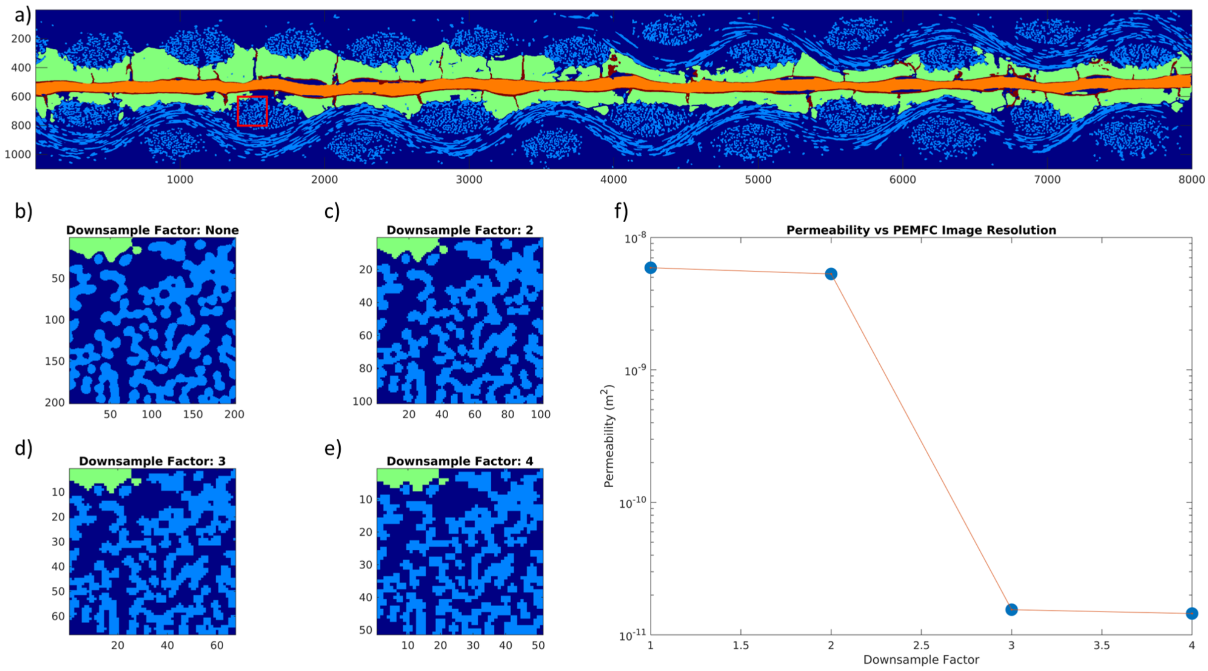

Figure 11: Analysis of the permeability and image detail vs the image resolution of the PEMFC. A slice (a) shows in zoomed sections (b-e) that a downsample factor of 2 retains overall flow connectivity and pore space detail to a degree, but both visual detail and (f) computed permeability fail at factors of 3 or more. The need for both a wide field-of-view as well as a high-resolution (and segmentable) image is critical in flow analysis.
