# Supplementary material for: Large-scale physically accurate modelling of real proton exchange membrane fuel cell with deep learning
Source: Nat Commun. 2023 Feb 14;14:745. doi: 10.1038/s41467-023-35973-8 (PMC9929041; doi:10.1038/s41467-023-35973-8)
Supplement: Supplementary file 3 — Description to Additional Supplementary Information [file 41467_2023_35973_MOESM3_ESM.pdf]

Description of Additional Supplementary Files

Supplementary Video: 3D animated render of water management. LBM simulations of water management within the super-resolved and multi-label segmented PEMFC during Free-Flow, Dead-Ended, and Purge operating conditions. The influence of MPL, GDL, and gas channel geometry and alignments are visually apparent, and the wide FOV with fully resolved micro-scale structures allows capturing of non-local flow phenomena.
